# Deletion Xq27.3q28 in female patient with global developmental delays and skewed X-inactivation

**DOI:** 10.1186/1471-2350-14-49

**Published:** 2013-05-01

**Authors:** Lauren S Marshall, Julie Simon, Tim Wood, Mei Peng, Renius Owen, Gary S Feldman, Michael V Zaragoza

**Affiliations:** 1 Pediatrics Department, Genetics & Metabolism Division, University of California, Irvine, Irvine, CA, USA; 2 Greenwood Genetic Center, Biochemical Genetics Laboratory, Greenwood, SC, USA; 3 Quest Diagnostics Nichols Institute, San Juan Capistrano, CA, USA; 4 Stramski Children’s Developmental Center, Miller Children's Hospital, Long Beach, CA, USA; 5 University of California, Irvine School of Medicine, 2042 Hewitt Hall, Irvine, CA, 92697-3940, USA

**Keywords:** X chromosome, X-inactivation, Chromosome deletion, Fragile X syndrome, Mucopolysaccharidosis II, X-linked mental retardation

## Abstract

**Background:**

Global developmental delay and mental retardation are associated with X-linked disorders including Hunter syndrome (mucopolysaccharidosis type II) and Fragile X syndrome (FXS). Single nucleotide mutations in the iduronate 2-sulfatase (*IDS*) gene at Xq28 most commonly cause Hunter syndrome while a CGG expansion in the *FMR1* gene at Xq27.3 is associated with Fragile X syndrome. Gene deletions of the Xq27-28 region are less frequently found in either condition with rare reports in females. Additionally, an association between Xq27-28 deletions and skewed X-inactivation of the normal X chromosome observed in previous studies suggested a primary role of the Xq27-28 region in X-inactivation.

**Case presentation:**

We describe the clinical, molecular and biochemical evaluations of a four year-old female patient with global developmental delay and a hemizygous deletion of Xq27.3q28 (144,270,614-154,845,961 bp), a 10.6 Mb region that contains >100 genes including *IDS* and *FMR1*. A literature review revealed rare cases with similar deletions that included *IDS* and *FMR1* in females with developmental delay, variable features of Hunter syndrome, and skewed X-inactivation of the normal X chromosome. In contrast, our patient exhibited skewed X-inactivation of the deleted X chromosome and tested negative for Hunter syndrome.

**Conclusions:**

This is a report of a female with a 10.6 Mb Xq27-28 deletion with skewed inactivation of the *deleted* X chromosome. Contrary to previous reports, our observations do not support a primary role of the Xq27-28 region in X-inactivation. A review of the genes in the deletion region revealed several potential genes that may contribute to the patient’s developmental delays, and sequencing of the active X chromosome may provide insight into the etiology of this clinical presentation.

## Background

Hunter syndrome (mucopolysaccharidosis type II; MIM 309900) and Fragile X syndrome (FXS; MIM 300624) are two X-linked disorders that can cause developmental delays and are infrequently found in females. In Hunter patients, dysfunction or absence of the iduronate-2-sulfatase (IDS) enzyme leads to the accumulation of mucopolysaccharides heparin sulfate and dermatan sulfate [1]. Single nucleotide mutations in the iduronate 2-sulfatase (*IDS*) gene at Xq28 most commonly cause Hunter syndrome but deletion of the *IDS* gene has been reported in Hunter patients [2-4]. Fragile X syndrome is usually associated with the expansion of the CGG trinucleotide repeat in *FMR1* at Xq27.3 region [5,6], deletion of *FMR1* has also been reported in males with Fragile X [7-10].

The Xq27-28 region, which contains *IDS* and *FMR1*, may also play a role in X-inactivation. Previous studies reported skewed X-inactivation in females with mental retardation and deletions within this region [11-15]. In this report, we describe a female patient with global developmental delays and a ~10.6 Mb deletion at Xq27.3-q28 (144,270,614-154,845,961 bp), a region that contains more than 100 genes including *IDS* and *FMR1*. X-inactivation studies and IDS activity assays were performed to determine the cause of her condition. Contrary to previous reports, our results do not support a role of the Xq27-28 region in X-inactivation.

## Case presentation

### Clinical summary

The patient is a four year-old female who presented at age 23 months to the Medical Genetics clinic with global developmental delays of unknown cause. She was born to a 29-year-old gravida IV, para III mother (x1 spontaneous abortion) and a 36-year-old father, both of Mexican ethnic background. At three months gestation her mother had a cholecystectomy and received morphine. At full term gestation, the patient was born by normal spontaneous vaginal delivery with no complications and was discharged the next day. Her birth weight was 7 lbs and 9 ounces (50th-75th percentile) and birth length was 21 inches (90th percentile).

The parents report that her delays in development were first noticed around age five months. At age nine months, the infant was diagnosed with global developmental delay and hypotonia. She began to crawl at 12 months and to sit on her own at 15 months. At the age of 22 months, neurological and formal developmental assessments were conducted at the Stramski Children’s Developmental Center in Long Beach, CA. The patient was assessed using the Bayley Scales of Infant Development (BSID III) and the relative chronological ages for the following developmental domains were as follows: cognitive (11 months), receptive language (3 months), expressive language (6 months), fine motor (15 months), and gross motor (11 months). The patient did not meet the diagnostic criteria for autism.

**Figure 1:**
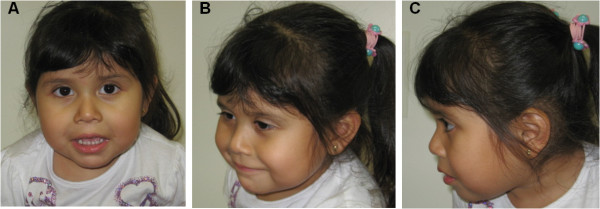
**Facial Features.** Front **(A)**, three-quarter **(B)**, and profile **(C)** views of the patient.

At age 23 months old, she was evaluated in the Medical Genetics clinic (MVZ and JS). Physical examination (Figure 1) showed that the patient had wide eye fissures, down turned corners of the lips, full cheeks, temporal narrowing, gaps between her teeth and long eyelashes. Her fingers were tapered with a hypoplastic distal crease of her left index finger, and she wore corrective lenses for myopia. She had limited speech and mild central hypotonia. Detailed pedigree analysis showed that she has healthy parents, two older siblings (a 14-year-old maternal half-brother and a 3-1/2-year-old full sister), and two paternal first cousins with autism and Asperger syndrome. There was no known consanguinity.

At the age of 31 months, follow-up development assessment by HELP Strands tests suggested global delays. The age equivalence was determined in the following skill domains: cognition (11–16 months), receptive language (8–13 months), expressive language/communication (6–9 months), gross motor (13–15 months), fine motor (13–16 months), social emotional (9–12 months), and self health (12–17 months). She was re-tested using BSID III and exhibited global development delays in both cognitive (percentile rank= 1) and language abilities (percentile rank= 0.1). At 31 months her relative chronological ages for the following developmental domains were as follows: cognitive (18 months), receptive language (9 months), expressive language (9 months), fine motor (20 months), gross motor (15 months).

Additional clinical evaluations included a normal electroencephalogram and brain MRI studies at age 31 months, normal electrocardiogram and echocardiography studies at age 3 years 5 months, and normal bone contour and density by radiographical skeletal survey at age 3 years 6 months.

At her last follow-up clinic visit at age 4 years-9-months old, the patient is making progress and no history of seizures or regression. Her parents report that recently she has displayed a greater sense of understanding when others communicate with her. She also points and uses her hands to communicate when she wants something. She has mild muscular hypotonia and her growth parameters are normal for her age.

### Molecular and biochemical studies

To investigate a genetic cause for the patient’s global developmental delays, blood chromosome analysis, DNA analysis for Fragile X syndrome, and Clarisure BAC array comparative genomic hybridization (CGH) studies were performed at Quest Diagnostics (San Juan Capistrano, CA). Initial biochemical laboratory studies included quantitative serum lactate, pyruvate and creatine phosphokinase (CPK), quantitative urine organic acids and plasma amino acids at St. Louis University Metabolic Screening Lab (St. Louis, MO). Urine biochemical studies for mucopolysaccharides (MPS) were performed at Quest Diagnostics (San Juan Capistrano, CA) and the Greenwood Genetic Center (Greenwood, South Carolina). IDS enzymatic assay and X-inactivation study of blood samples were performed by the Greenwood Genetic Center. The androgen receptor locus (*HUMARA*) at Xq12 was used to study X-inactivation [16]. The ratio of active to inactive X chromosome was determined by PCR analysis examining DNA methylation-sensitive restriction enzyme sites near the polymorphic CAG repeat in first exon of *HUMARA*[16]. Molecular studies for the patient’s parents included Fragile X PCR and Southern Blot analysis and Oligonucleotide SNP array studies and were performed by Quest Diagnostics (San Juan Capistrano, CA). Details of the BAC and Oligonucleotide SNP arrays are available at http://www.questdiagnostics.com.

#### Deletion region detected at Xq27.3q28

**Figure 2:**
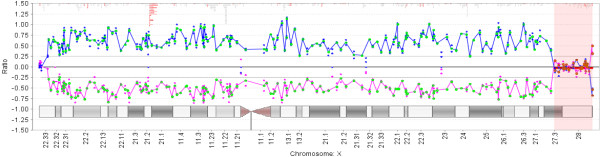
**Array CGH ratio plot depicts ~10.6 Mb at Xq27.3-q28.** Deletion region is highlighted in red. The deletion resulted in hemizygosity for >100 genes including *FMR1* and iduronate 2-sulfatase (*IDS*). BAC probes (CTD-3109C8 to CTD-2341N11) targeting multiple loci were used. Array was performed using Clarisure CGH by Quest Diagnostics.

**Figure 3:**
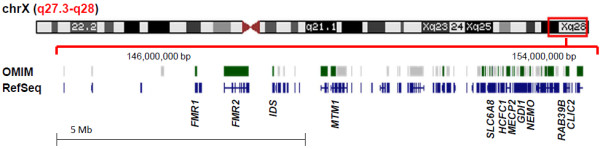
**Schematic of X chromosome and genes in the Xq27.3**-**q28 region.** Region of deletion is highlighted by the red box. Shown are the OMIM-disease associated genes in green and all RefSeq genes in blue within the deletion region of 144,270,614-154,845,961 bp (hg19). Image was adapted from http://genome.ucsc.edu/cgi-bin/hgTracks.

Chromosome and DNA studies found an abnormal karyotype with a deletion on the X chromosome. Fragile X PCR revealed only one unmethylated *FMR1* allele within the normal size range (~30 CGG repeats), and Southern Blot analysis confirmed a deletion of the *FMR1* region on one X chromosome. BAC array showed a ~10.6 megabase deletion at Xq27.3q28 (144,270,614-154,845,961 bp) encompassed within BAC clones CTD-3109C8 to CTD-2341N11 (Figure 2). This deletion resulted in hemizygosity for 113 RefSeq genes including the *FMR1* and iduronate 2-sulfatase (*IDS*) genes which are associated with Fragile X syndrome (FXS) and Hunter syndrome, respectively (Figure 3). OMIM disease-associated genes within the deletion region included: *FMR1*, *AFF2* (*FMR2*), *IDS*, *MAMLD1*, *MTM1*, *NSDHL*, *ATP2B3*, *FAM58A*, *SLC6A8*, *ABCD1*, *L1CAM*, *AVPR2*, *NAA10*, *HCFC1*, *MECP2*, *OPN1LW*, *OPN1MW*, *FLNA*, *EMD*, *RPL10*, *TAZ*, *GDI1*, *G6PD*, *IKBKG* (*NEMO*), *DKC1*, *F8*, *RAB39B*, *CLIC2*, and *TMLHE*.

Parental *FMR1* PCR and Southern Blot analysis revealed ~30 and 31 repeats for the mother and ~31 repeats for the father. Oligonucleotide SNP arrays were normal for both parents. These findings indicate that the deletion was a *de novo* event (Table 1).

**Table 1:** Summary of results

**Test**	**Patient**	**Father**	**Mother**
Array^*^	Xq27.3q28 (CTD-3109C8 > CTD-2341N11) x1	Normal	Normal
***FMR1***	30 repeats	31 repeats	30 and 31 repeats
**IDS activity**	Normal	n.d.	n.d.
**X**-**inactivation**	100:0 ratio	n.d.	n.d.

*Based on BAC array for patient and Oligonucleotide SNP array for parents; n.d., not done.

#### Skewed X-inactivation studies and negative biochemical studies for Hunter syndrome

Normal results were obtained for serum lactate, pyruvate and creatine phosphokinase (CPK), quantitative urine organic acids and plasma amino acids. Initial quantitative urine analysis found elevated levels of mucopolysaccharides (17.5 mg/mmol creatinine; reference range= <16.0 mg mg/mmol creatinine) at age 3 years-old. However, repeat urine studies at age 3 years 10 months-old found normal quantitative (6.97, normal less than 16) and qualitative urine MPS studies.

The peripheral blood samples tested normal for IDS activity (808 nmol 4MU released/hr/ml plasma; normal range= 182–950 4MU released/hr/ml plasma). The HUMARA X-inactivation studies showed two alleles with PCR lengths of 287 and 299 base pairs that correspond to CAG repeat lengths of 22 and 26 repeats, respectively. Methylation analysis showed that the 287 PCR fragment (22 repeats) was methylated corresponding to an X-inactivation ratio of 100:0, indicating highly skewed X-inactivation. Because *IDS* is found within the deletion region and we assume that our patient only has one functional copy of the gene, then the functioning *IDS* gene must be on the preferentially active X chromosome and the deletion region must be on the inactive X chromosome. Therefore, we concluded that the X chromosome with the deletion region was preferentially inactivated (Table 1).

## Discussion

We report a four year-old female patient with global developmental delay, hypotonia, and a ~10.6 Mb hemizygous deletion of Xq27.3q28. To determine the specific cause of her condition, we conducted biochemical and X-inactivation studies on the proband and molecular studies on the parents. There are over 100 genes (113 RefSeq and 29 OMIM disease-associated genes) currently described within the deletion region, and we considered a number of the associated conditions and the potential role of unbalanced X-inactivation as a genetic mechanism for our patient’s condition.

Hunter syndrome was initially considered as a potential diagnosis because the iduronate 2-sulfatase (*IDS*) gene was located in the deletion region and if it did turn out that Hunter syndrome was the cause of her condition, we could offer our patient therapy through enzyme replacement. *IDS* deletions are found in some Hunter syndrome patients [2-4,17], and a more severe Hunter phenotype has been reported in patients with larger deletion regions that include *FMR1*[17]. Additionally, it was found that a female Xq27-28 deletion patient with Hunter syndrome [11] had unbalanced X chromosome inactivation in which the normal X chromosome was preferentially inactivated. However, unlike Clarke’s patient [11], our patient lacked the typical features for storage diseases including macrocephaly, coarse features, hepatomegaly, cardiac and skeletal abnormalities [18]. Nevertheless, we pursued the diagnosis when her initial urine analysis revealed a mild elevation of mucopolysaccharides. However, repeat studies found normal mucopolysaccharide levels and distribution and IDS enzymatic assay in blood leukocytes was normal. Thus, the diagnosis of Hunter syndrome was excluded in our patient. Additionally, the positive IDS assay suggested that the functional IDS gene was located on the active X chromosome; therefore we made the assumption that the deletion region was located on the inactive X chromosome.

We next considered other X-linked mental retardation conditions including Fragile X syndrome as the *FMR1* gene is also located in the deletion region and *FMR1* deletions are associated with Fragile X syndrome [7-10]. Following Clarke *et al*., 1992, at least three additional deletion Xq27 or Xq27-28 females with hemizygosity for *FMR1* have been reported [12-15]. Similar to our patient, these females had non-specific characteristics of Fragile X syndrome such as hypotonia, speech, motor, and language delays, and cognitive impairment [19]. Two of the patients also had unbalanced X-inactivation of the normal X chromosome; thus, at least partial loss of *FMR1* or other genes within the deletion may contribute to their phenotype [13-15]. However, in contrast to these previous reports, our patient had preferential inactivation of the deleted X-chromosome and a normal sized and unmethylated *FMR1* allele, and the diagnosis of Fragile X syndrome was also excluded.

It is possible that the large size of the deletion in our patient may explain the highly skewed inactivation pattern. Compared to previous Xq27-28 deletion females [12], the deletion of our patient extends far beyond *IDS* including practically all of Xq28. Highly skewed inactivation pattern has been observed in patients with gene mutations within the Xq28 region due to negative cell selection. For example, skewed inactivation has been reported in rearrangement and truncation mutations that result in a loss of function in the *NEMO* (*IKBKG*) gene, which is associated with incontinentia pigmenti, hypohidrotic ectodermal dysplasia, and other types of immunodeficiencies [20-22]. In our patient, negative selection of cells expressing the mutant *NEMO* allele on the deleted X-chromosome may then result in the highly skewed X-inactivation; however, this alone cannot explain her clinical phenotype.

Additionally, significant variation in X-chromosome inactivation patterns have been found in different tissue types [23,24]. In particular, in patients with differences between tissues, peripheral blood was consistently skewed with greater than 75% expression of one chromosome [23]. Therefore, our X-inactivation analysis on blood leukocytes may not be representative of all tissues, and we could consider additional X-inactivation studies on other tissue types in order to better understand our patient’s gene expression profile.

Another aspect of X-inactivation to consider is whether any of the genes implicated in our patient’s condition escape X-inactivation. Approximately 10% of X-linked genes show variation in escape *in vitro* and a cluster of these genes is found in the Xq28 region [25]. If some of the genes that normally escape X-inactivation are within our patient’s deletion region, then haploinsufficiency could account for our patient’s observed phenotype. Several genes within the Xq28 region appear to escape X-inactivation, but we found that none of these genes are related to our patient’s phenotype [25]. Furthermore, Carrel and Willard (2005) found that *IDS*, *MTM1*, and *MECP2* were not expressed from any of the inactivated X chromosomes, and *FMR1* was expressed in only one out of nine inactive X chromosome samples [25]. Therefore, it does not appear as though that the deletion of these genes would have an adverse effect since they would not normally be expressed from an inactivated X chromosome. Nevertheless, the extent to which these findings can be applied to our case study is somewhat limited as the results were obtained *in vitro* from fibroblast cells and may not reflect the *in vivo* expression or expression in different tissues.

A final alternative cause of our patient’s phenotype may be an unknown, additional mutation on the active normal X-chromosome. Over 100 genes on the X chromosome have been associated with syndromic and non-syndromic X-linked intellectual disabilities [26]. An estimated 40% of the protein-coding genes on the X chromosome are expressed in the brain and mutations in these genes have the potential to cause mental retardation [27]. In addition to *IDS* and *FMR1*, a literature review revealed several genes within our patient’s deletion region- *SLC6A8*, *MECP2*, *GDI1*, *CLIC2*, *HCFC1*, *RAB39B*, and *MTM1*- that play a role in physical and mental development. Hahn *et al*. (2002) report that the mutation of the creatine-transporter (*SLC6A8*) gene was found in patients with mild retardation and other behavioral problems, and Rosenberg *et al*. (2004) described one patient with X-linked mental retardation that coincided with a large deletion of the *SLC6A8* and other patients with missense mutations of the gene [28,29]. Partial or complete loss of function mutations of the *MECP2* gene has been associated with mental retardation and Rett syndrome [30]. Other X-linked forms of mental retardation have been reported in patients with mutations in *GDI1*[31], *CLIC2*[32], *HCFC1*[33], and *RAB39B*[34]. The deletion of *MTM1* gene may be associated with our patient’s hyptonia as *MTM1* deletions and mutations have been shown to be related to myotubular myopathy [35]. Additionally, premature ovarian failure has been reported in patients with deletions within the Xq26.2-q28 region [36], and will be important to consider as part of the long-term care of our patient.

Based on the highly skewed X-inactivation pattern observed in our patient, mutations in the active X chromosome are especially important to consider as potential causes of her etiology. Specific conditions and their associated genes in Xq27-28 that require investigation include FRAXE syndrome- *FMR2*, Rett syndrome-*MECP2*, X-linked Mental retardation-41-*GDI1*, Creatine deficiency syndrome-*SLC6A8*, X-linked mental retardation-72-*RAB39B* and X-linked adrenoleukodystrophy-*ABCD1*.

## Conclusions

In summary, we report a female with global developmental delay, hypotonia, and a ~10.6 Mb hemizygous deletion of Xq27.3q28. Biochemical, molecular and X-inactivation studies failed to support the diagnosis of Hunter syndrome or Fragile X syndrome. In contrast to previously reported deletion females, the *deleted* X-chromosome was preferentially inactivated. Our results do not support a primary role of the Xq27-28 region in X-inactivation. In addition, given that this region is often involved in genomic rearrangements, we might speculate that the breakpoint region may contain highly repetitive DNA sequences that predispose to abnormal recombination [37]. Future studies may include high-throughput sequencing of the normal X chromosome, analysis of the deletion breakpoints, and X-inactivation studies on different tissues to understand the specific mechanism for the expression of an X-linked disease in a deletion carrier female.

### Consent

Written informed consent was obtained in accordance with the UC Irvine Institutional Review Board for human subject research. The parents gave permission for publication of the case, their own clinical details and for the publication of the images. A copy of the written consent is available for review by the Series Editor of this journal.

## Competing interests

The authors declare no competing financial interests.

## Authors’ contributions

GSF, LSM, JS, and MVZ identified the patient and carried out the clinical evaluations; TW, MP, and RO conducted the molecular and biochemical studies and analyzed the results; LSM and MVZ wrote the manuscript. All authors read and approved the final manuscript.

## Pre-publication history

The pre-publication history for this paper can be accessed here:

http://www.biomedcentral.com/1471-2350/14/49/prepub
